# Comparative study of acute kidney injury in pararenal aortic aneurysm: open surgical versus endovascular repair

**DOI:** 10.3389/fsurg.2024.1457583

**Published:** 2024-09-10

**Authors:** Sherif Sultan, Yogesh Acharya, Wael Tawfick, William Wijns, Osama Soliman

**Affiliations:** ^1^ Department of Vascular and Endovascular Surgery, Western Vascular Institute, University Hospital Galway, University of Galway, Galway, Ireland; ^2^ Department of Vascular Surgery and Endovascular Surgery, Galway Clinic, Royal College of Surgeons in Ireland and University of Galway, Galway Affiliated Hospital, Doughiska, Ireland; ^3^ CORRIB-CURAM-Vascular Group, University of Galway, Galway, Ireland; ^4^ The Euro Heart Foundation, Amsterdam, Netherlands

**Keywords:** pararenal abdominal aortic aneurysm, endovascular aneurysm repair, open surgical repair, acute kidney injury, comparative study

## Abstract

**Background:**

Pararenal abdominal aortic aneurysms (PR-AAA), constituting around 15%-20% of AAA patients, are defined as having no neck between the aneurysm and the renal arteries. Due to an insufficient sealing zone, open surgical repair (OSR) is the gold standard, while EVAR is reserved for those unfit for surgery. Renal outcomes disturb long-term survival, and they have massive socioeconomic and quality of life implications, especially if patients require dialysis.

**Methods:**

This study aims to elucidate any difference between EVAR and OSR of PR-AAA, excluding suprarenal aneurysms, with specific emphasis on renal dysfunction over the short and long term. An existing database of PR-AAA between 2002 and 2023 was used to glean information regarding the therapeutic option used. Renal events were defined by the RIFLE criteria. Out of 1,563 aortic interventions, we identified 179 PR-AAA, of which 99 high-risk patients had an aortic neck of less than 10 mm with complete follow-up. We excluded patients with fenestrated EVAR (FEVAR), branched EVAR (BEVAR), or chimney EVAR (Ch-EVAR) and any patients requiring visceral artery reimplantation.

**Results:**

In total, 63 patients underwent EVAR, and 36 required OSR. 17.46% of patients who underwent EVAR experienced acute kidney injury (AKI) compared with 36.11% of the OSR group (*P* = 0.037). The mean post-op creatinine for OSR was 109.88 µmol/L, and for EVAR was 127.06 µmol/L (*P* = 0.192). The mean difference between long-term (9–12 years) creatinine values in OSR was 14.29 µmol/L (*P* = 0.191), and the mean difference for EVAR was 25.05 µmol/L (*P* = 0.024). Furthermore, 27.8% of OSR patients who underwent Left Renal Vein Division and Ligation (LRVDL) experienced an AKI, while 50% who did not undergo LRVDL experienced an AKI (*P* = 0.382). Thirty-day morbidity in the EVAR group (20.97%) was significantly lower than in the OSR group (42.62%) (*P* = 0.022). Moreover, 3.17% in EVAR group and 7.14% in OSR group had aneurysm-related mortality (*P* = 0.584).

**Conclusion:**

The rate of renal events for OSR is higher, while the rate of endovascular renal events was lower. Our study shows that PR-AAA undergoing OSR may benefit from endovascular repair.

## Introduction

Pararenal abdominal aortic aneurysms (PR-AAA), subdivided into juxta-renal AAA (JR-AAA) and suprarenal AAA (SR-AAA), account for approximately 15%-20% of AAAs (1, 2). JR-AAA aneurysms extend within 15 mm of the renal arteries without involving the renal artery origins, whereas suprarenal aneurysms involve them (2–4). JR-AAA are clamped superior to the renal arteries, and SR-AAAs or type IV are clamped superior to the celiac plexus or the superior mesenteric artery that requires renal revascularization (5).

PR-AAA presents a formidable challenge in the realm of vascular surgery, particularly among high-risk patients undergoing open AAA repair (1–5). Although complex AAAs are estimated to account for approximately 15%–20% of all AAAs, repairs of these complex AAAs carry a higher perioperative risk and are supported by limited evidence and studies (1–3). Also, there is considerable uncertainty regarding the effectiveness of open surgical repair (OSR) vs. endovascular strategies for complex AAAs. Central to the success of such procedures is the meticulous consideration of surgical techniques, notably the pivotal role played by left renal vein transfixing in optimizing exposure of the juxta-renal area (1–10).

Despite advancements in perioperative care, studies underscore the substantial incidence of acute kidney injury (AKI) following suprarenal clamping during AAA repair, highlighting the imperative for refined surgical methodologies (11, 12). Cross-clamping during OSR may cause renal dysfunction (5), while in endovascular aneurysm repair (EVAR) patients, contrast-induced nephropathy during fluoroscopy and microembolization during wire manipulation lead to renal ischemia (13–16).

AKI prolongs hospitalization and increases morbidities post-op (17, 18). Short and long-term renal dysfunction is higher after PR-AAA than infrarenal AAA repair (19, 20). OSR of JR-AAA is predictive of renal dysfunction (21–25). Prolonged renal ischemia time emerges as a significant factor predicted by intraoperative blood loss and AKI occurrence, necessitating precise perioperative management strategies (26).

Most published studies on complex AAAs are case studies, which are limited by selection and indication biases. These studies often focus on operative techniques rather than detailed anatomical considerations, pooling data from various types of aneurysms, including short-neck, juxta-renal, pararenal, para-visceral, and thoracoabdominal aortic aneurysms. Therefore, this real-world clinical study presents key findings to underscore the importance of expeditious proximal anastomosis creation and optimal clamp positioning in minimizing renal morbidity and mortality risks during PR-AAA repair, particularly in patients with compromised renal function**.** Moreover, it scrutinizes standard EVAR as a bail-out in endovascular armamentarium in high-risk patients.

### Objectives

This study aims to elucidate any difference between EVAR and OSR for treating patients with PR-AAA, excluding SR-AAAs, with specific emphasis on renal dysfunction over the short and long term.

Our primary outcome is AKI. Similarly, our secondary outcomes are renal failure, left renal vein division and ligation (LRVDL), chronic kidney disease (CKD), aneurysm-related mortality and all-cause mortality.

## Methods

### Patients

All patients who underwent elective surgery for JR-AAA repair in a tertiary referral centre from October 2002 to December 2023 were included in the study. We had 9,937 referrals with a diagnosis of AAA, out of which 1,563 had aortic intervention (1,176 endovascular vs. 387 OSR). Amongst them, we identified and scrutinized 179 pararenal aneurysms, of which 99 patients had an aortic neck of less than 10 mm and had complete follow-up. We excluded patients who had fenestrated EVAR (FEVAR), branched EVAR (BEVAR), or chimney EVAR (ChEVAR) or required visceral artery re-implantation, i.e., type IV or SR-AAA. Moreover, patients who underwent an emergency procedure for rupture or stenting of the aorta for aorto-iliac disease were excluded.

### Data collection

The project was approved by our institutional clinical research ethics committee (C.A.2635). Demographics and outcomes were reported according to the Society for Vascular Surgery guidelines.

Clinical, operative, and radiological data were collected from a prospectively maintained database: Vascubase™ (Version 5.2, Consensus Medical Systems, INC. Richmond, British Colombia, Canada). Patients’ medical notes were reviewed to complete the required clinical data. An existing database of pararenal aneurysms between 2002 and 2023 was used to glean information regarding the therapeutic option used.

### Procedure

All patients were evaluated with duplex ultrasound. Those with AAAs of at least 4.5 cm were further evaluated with computed tomographic angiography (CTA).

The threshold for intervention in asymptomatic men was 5.5 cm and 5 cm in women. Symptomatic patients were treated if the AAA expanded by 0.5 cm in 6 months or if the AAA was saccular or eccentric in shape.

OSR or EVAR was offered as appropriate. However, the decision on which treatment modality to use for each patient was informed by life expectancy, operative risk, the risk of rupture, and the patient's wishes. When patients were treated with EVAR outside of the instructions for use (IFU), they were fully aware of the plan and its associated risks and benefits.

Preoperatively, patients were worked up with electrocardiography, echocardiography, and chest radiography. Ankle-brachial pressure index (ABPI) and carotid Doppler's were performed on each patient to establish peripheral vascular disease and carotid stenosis. Baseline laboratory testing included d-dimer, serum N-terminal pro-B-type natriuretic peptide, troponin T, and creatinine kinase.

### Open surgical repair (OSR)

Patients undergoing OSR were administered intravenous saline, N-acetylcysteine, 20% mannitol, antibiotics, and heparin. The aorta was exposed via a trans-peritoneal approach. The left renal vein was dissected close to the origin at the inferior vena cava in cases where the neck of the aorta could not be sufficiently exposed. We preserved the suprarenal and gonadal branches. Cross-clamping was applied suprarenal or diagonally, sparing the more superior renal artery where possible. Silver-impregnated Dacron grafts (Intervascular, Data scope Corp., Fairfield, NJ, USA) were used as the standard, with a tube or bifurcated models employed depending on the Iliac anatomy. The proximal graft was spatulated to incorporate the renal arteries into the suture line, where there was a healthy aorta at the renal artery level. Where there was no healthy aorta at the level of the renal arteries, the renal artery was re-implanted with a Carrel patch. Cell saver re-transfusion was used routinely, and patients were transferred to the intensive care unit (ICU) postoperatively.

Patients who underwent OSR were reviewed in the outpatient clinic at six weeks post-op, then six monthly for 18 months, and then yearly. ABPIs were completed at each visit. CTA was performed routinely at five years and before that time if indicated.

### Endovascular aneurysm repair (EVAR)

The devices used for EVAR were selected based on each patient's anatomy. These devices included: Aorto-Uni-Iliac (AUI) Medtronic or Talent (Medtronic, Santa Rosa, CA, USA), Medtronic Endurant, Bifurcated Medtronic Talent, Bifurcated Medtronic Endurant, Bifurcated Gore Excluder (W.L. Gore & Associates, Flagstaff, AZ, USA), Unibody Endologix AFX and Powerlink (Endologix, Irvine, CA, USA) and Cordis Incraft (Cordis, a Johnson & Johnson company, Miami Lakes, FL, USA). The bifurcated aorto-bi-iliac approach was used for standard EVAR, and the aorto-uni-iliac approach was used for unfavorable anatomy (three cases). Proximal endografts were oversized by 24%–30%. Depending on the anatomy and pathology of the individual patient, 36-mm Endurant cuffs or Palmaz giant stent (Cordis, a Johnson & Johnson company, Miami Lakes, FL, USA) were used as a DRESS Technique (27).

Before discharge, EVAR patients had biplanar abdominal radiography, ABPI, and colour duplex ultrasound. These investigations were repeated at six weeks, three months, six months, and biannually after that. CT was only performed if the duplex ultrasound showed sac expansion or endoleak.

### Renal dysfunction

AKI was defined using the RIFLE (Risk, Injury, Failure, Loss of kidney function, and End-stage kidney disease) criteria (28). CKD is the progressive loss of nephrons resulting in permanent loss of renal function. CKD can be estimated according to glomerular filtration rate (GFR) by using the modification of diet in renal disease equation (MDRD) (29). The changes to the baseline must be present for three months to differentiate CKD from AKI. Unless there is evidence of kidney damage as specified in the medical notes, stage three CKD was used as the cut-off for CKD (30).

### Statistical analysis

The IBM Statistical Package for the Social Sciences (SPSS) Statistics Version 22 (IBM Corp., Armonk, NY, USA) was used for data analysis. Mean, standard deviation (SD) or median, interquartile range (IQR) were used to report on continuous data depending on the normality of distribution. Pearson's chi-squared test, Fisher's exact test, independent sample *t*-test, Mann-Whitney *U*-tests, and log-rank were used as necessary.

## Results

In total, 63 patients underwent EVAR, and 36 required OSR. Table 1 gives baseline demographics.

**Table 1:** Baseline characteristics of the patients.

Demographics	EVAR^a^ (*n* = 63)	OSR^b^ (*n* = 36)	*P*-value
Age, years	74.79 ± 9.314	73.28 ± 73.78	*P* = 0.404
Male, *n*	48 (76.20%)	27 (75.00%)	*P* = 0.849
Abdominal aortic aneurysm (AAA) size, mm	5.8595 ± 1.66	7.08 ± 2.01	*P* = 0.002*
Juxta-renal (<15 mm short of renal ostia)	53 (84.13%)	28 (77.80%)	*P* = 0.431
Pre-operative chronic kidney disease (CKD), *n*	26 (41.30%)	16 (44.44%)	*P* = 0.759
Hyperlipidaemia, *n*	37 (68.80%)	19 (59.38%)	*P* = 0.830
Diabetes Mellitus, *n*	10 (16.00%)	7 (19.44%)	*P* = 0.676
Current Smoking, *n*	16 (25.40%)	12 (34.29%)	*P* = 0.319
Peripheral Vascular Disease, *n*	8 (13.00%)	3 (8.33%)	*P* = 0.741
Coronary artery disease, *n*	24 (38.10%)	9 (25%)	*P* = 0.166
Carotid artery disease, *n*	3 (4.80%)	5 (13.89%)	*P* = 0.139
Hypertension, *n*	43 (68.30%)	28 (68.57%)	*P* = 0.844
Antiplatelet, *n*	8 (13.00%)	4 12.5%	*P* = 0.910
Calcium channel blocker (CCB), *n*	12 (19.10%)	1 (3.13%)	*P* = 0.030*
Beta Blocker, *n*	24 (38.10%)	17 (53.13%)	*P* = 0.228
Anticoagulant, *n*	38 (60.32%)	14 (43.75%)	*P* = 0.071
ACE-i or ARB^c^, *n*	20 (31.803%)	10 (31.25%)	*P* = 0.893
Statin, *n*	38 (60.32%)	19 (59.38%)	*P* = 0.710

^a^ Endovascular Aneurysm Repair.

^b^ Open surgical repair.

^c^ Angiotensinogen Converting Enzyme inhibitors or Angiotensin receptor blocker.

* Signifies significant results.

There was no significant difference in gender (*P* = 0.849), age (*P* = 0.404), and preoperative comorbidities and medications between the EVAR and OSR groups. However, the mean aneurysm diameter for patients undergoing OSR was significantly larger than the EVAR groups (7.08 ± 2.01 vs. 5.8595 ± 1.66; *P* = 0.002).

The mean intraoperative time for EVAR was 3.04 ± 1.18 h, while OSR was 3.25 ± 1.03 h (*P* = 0.208). In total, 6.35% of patients undergoing EVAR required red cell transfusion compared with 50% of OSR patients (*P* = 0.0003).

Amongst the OSR group, 2 had a retro-aortic left renal vein; both were transfixed before clamping. Overall, 27.8% of patients who underwent LRVDL experienced an AKI. No patient underwent left renal vein reimplantation reconstruction, as this method is not used in our institution.

### Peri-operative morbidity and mortality

Thirty-day morbidity in the EVAR group was significantly lower than in the OSR group (20.97% vs. 42.62%; *P* = 0.022) (Table 2). Also, 3.23% of patients in the EVAR group experienced myocardial infarction compared to 13.89% in the OSR group (*P* = 0.096). No patients from either group experienced a stroke at 30 days, deep vein thrombosis or pulmonary embolism, and one patient that underwent OSR experienced bowel ischemia.

**Table 2:** Perioperative complications following standard endovascular aneurysm repair (EVAR) versus open surgical repair (OSR) in patients with pararenal abdominal aortic aneurysm.

Post-op complications	EVAR (*n* = 63)	OSR (*n* = 36)	*P*-Value
Renal failure	2 (3.17%)	2 (5.56%)	*P* = 0.623
Stroke	0	0	*P* = 1.000
Dialysis	3 (4.76%)	2 (5.55%)	*P* = 0.609
Myocardial infarction	2 (3.17%)	5 (13.89%)	*P* = 0.096
Cardiac	3 (4.76%)	7 (19.44%)	*P* = 0.035
Respiratory	5 (7.94%)	5 (13.89%)	*P* = 0.358
Bowel ischaemia	0	1 (2.78%)	–
Morbidity 30 day	13 (20.63%)	15 (41.67%)	*P* = 0.022
Mortality 30 day	0	1 (2.78%)	–

Two patients (5.55%) required dialysis after OSR. Of these patients, one patient required long-term dialysis, and one required temporary dialysis for two weeks until recovering from acute kidney injury. Three (4.80%) patients required new-onset dialysis post-EVAR, two long-term and one temporary.

There was no 30-day mortality in the EVAR group. However, the OSR group had one perioperative mortality, a 75-year-old man who passed away on day 29 post-operative due to bowel ischemia and multi-organ system failure (MOSF). This patient had a previous history of right hemicolectomy.

### Long term follow-up

The overall time to the last follow-up in the EVAR group was 42.17 ± 32.38 months, and in the OSR group, 50.96 ± 38.8 months (*P* = 0.274) (Table 3). Compared with EVAR, patients in the OSR group had a significantly longer stay in ICU (mean five days vs. one day; *P* = 0.004) and high dependency unit (three days vs. one day; *p* = 0.001). Also, OSR patients had significantly longer total inpatient stays than EVAR (13 days vs. 5 days; *P* = 0.004).

**Table 3:** Long-term complications following standard endovascular aneurysm repair (EVAR) versus open surgical repair (OSR) in patients with pararenal abdominal aortic aneurysm.

Post 30-day up to last follow-up	EVAR (*n* = 63)	OSR (*n* = 36)	*p*-value
Cardiac	4 (6.35%)	7 (19.45%)	*P* = 0.066
Renal	8 (12.69%)	5 (13.89%)	*P* = 0.990
Pulmonary	4 (6.35%)	8 (22.22%)	*P* = 0.030
Deep Venous Thrombosis	0	0	–
Pulmonary Embolism	0	0	–
Cerebrovascular Accidents	0	3 (8.33%)	*P* = 0.053
Coagulopathy	10 (15.87%)	14 (38.89%)	*P* = 0.019
Systemic Complications	16 (25.39%)	23 (63.89%)	*P* = 0.001
Access site Infection	2 (3.17%)	1 (2.78%)	*P* = 1.000
Total hospital stay	6 (9.52%)	13 (36.11%)	*P* = 0.004
Intensive Care Unit	1 (1.59%)	5 (13.89%)	*P* = 0.004
High Dependency Unit	1 (1.59%)	3 (8.33%)	*P* = 0.000
Aneurysm related to Death	2 (3.17%)	2 (5.56%)	*P* = 0.584
Time to death (not aneurysm specific)	1,163.17 ± 785.96	1,517.53 ± 1,097.12	*P* = 0.219
Time to last follow-up	1,264.96 ± 971.5 (42 months)	1,529.18 ± 1,163.09 (51 months)	*P* = 0.274

The mean post-operative creatinine for OSR was 109.86 µmol/L, and for EVAR was 127.06 µmol/L (*P* = 0.279) (Table 4). The mean difference between long-term creatinine values in OSR was 14.29 µmol/L (*P* = 0.191), and the mean difference for EVAR was 25.05 µmol/L (*P* = 0.024). Paired samples *t*-test showed no difference between preoperative and long-term eGFR values for EVAR (*P* = 0.086) or OSR (*P* = 0.482). In total, 17.46% of patients who underwent EVAR experienced AKI compared with 36.11% of the OSR group (*P* = 0.037).

**Table 4:** Renal outcomes of the patients following standard endovascular aneurysm repair (EVAR) versus open surgical repair (OSR) in patients with pararenal abdominal aortic aneurysm.

Post-operative renal insufficiency	EVAR (*n* = 63)	OSR (*n* = 36)	*P*-Values
RIFLE^a^ total episodes	11 (17.46%)	13 (36.11%)	*P* = 0.037
Risk (x1.5)	3 (4.76%)	5 (13.89%)	*P* = 0.109
Injury (x2)	5 (7.94%)	6 (16.67%)	*P* = 0.184
Failure (x3)	3 (4.76%)	2 (5.56%)	*P* = 0.862
Temporary dialysis	1 (1.59%)	1 (2.78%)	*P* = 0.685
Long term dialysis	2 (3.17%)	1 (2.78%)	*P* = 0.912
Post-op chronic kidney Disease	27 (42.86%)	15 (41.67%)	*P* = 0.737
Pre-op mean creatinine, µmol/L	109.75	99.54	*P* = 0.213
Post-op mean creatinine, µmol/L	122.4	134.12	*P* = 0.409
Long-term mean creatinine, µmol/L	127.06	109.86	*P* = 0.279
Preop mean eGFR^b^, ml/min/1.73m^2^	63.38	65.06	*P* = 0.770
Long term eGFR^b^, ml/min/1.73m^2^	56.75	60.76	*P* = 0.675

^a^ RIFLE: risk of renal dysfunction, Injury to the kidney, Failure or loss of kidney function, and end-stage kidney disease.

^b^ eGFR: estimated glomerular filtration rate.

Regarding CKD, 44.44% of patients with OSR met the criteria for CKD pre-operatively and 41.67% after five years. Although there was no significant difference in post-op CKD between the endovascular and OSR groups (*P* = 0.737), there was a significant change in mean creatinine from baseline to 5-year follow in the OSR group (0.024).

During the long-term follow-up, there was no difference between groups for cardiac or renal events but a significantly higher number of pulmonary events for OSR (*P* = 0.030). During the same period, there were three cerebrovascular accidents within the EVAR group but none in OSR. There was also a significantly higher number of systemic complications (63.89% vs. 27.59%; *P* = 0.001) and coagulopathy (38.89% vs. 17.24%, *P* = 0.019) within the OSR group.

**Figure 1:**
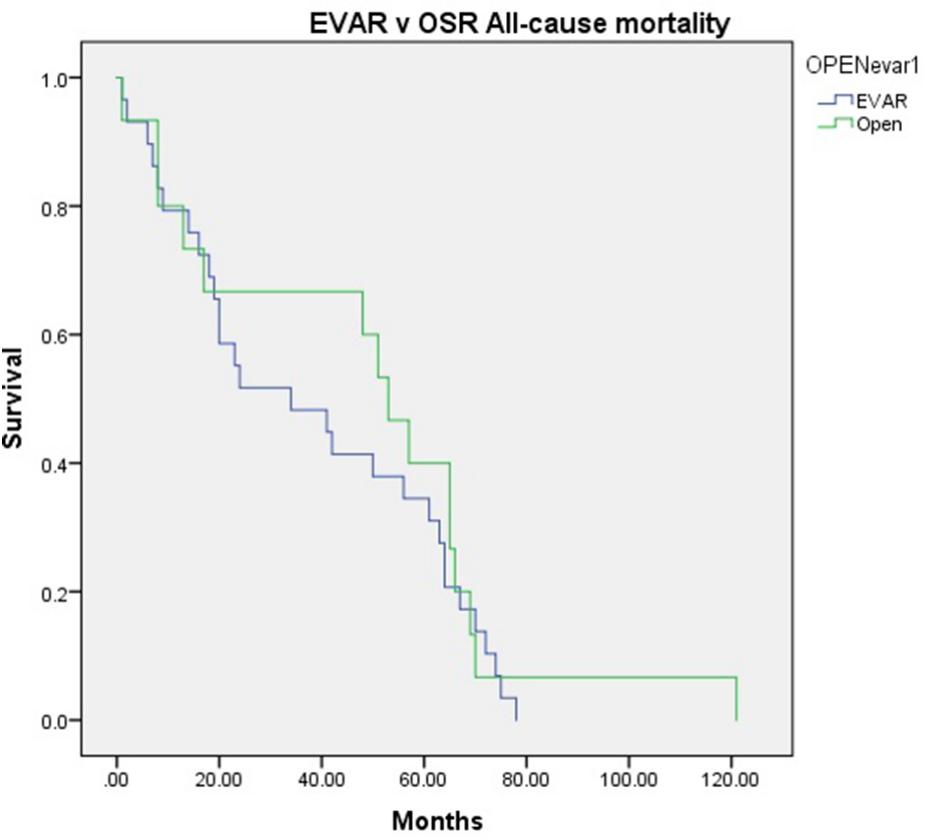
Kaplan-Meier curve showing the all-cause mortality following standard endovascular aneurysm repair (EVAR) versus open surgical repair (OSR) in patients with pararenal abdominal aortic aneurysm.

Kaplan Meier Curve shows the all-cause mortality in Figure 1. Two patients from the EVAR (3.17%) group and two from the OSR (7.14%) group died due to complications related to their aneurysm (*P* = 0.584).

In the EVAR group, one patient died due to septic shock, respiratory acidosis, and acute renal failure after OSR conversion due to his EVAR thrombosis seven months after the initial procedure. The second patient died seven years after the initial EVAR. This patient required no intervention due to graft infection post-streptococcal pneumonia with septicemia and was deemed to be unfit for reintervention.

In the OSR group, one patient experienced an AKI, MI, and respiratory complications perioperatively and required dialysis before he died 11 months later. The second OSR patient’ died at 29 days due to MOSF post-bowel ischemia. A log regression yielded no significant outcomes.

### Reinterventions

Twelve (19.05%) patients required a second intervention, all initially repaired by EVAR (*P* = 0.016). The most common reason for reintervention was type 1A endoleak for severe angulation of graft (6 patients), left limb thrombosis (4 patients), and two patients with type III endoleaks.

Over the follow-up period, of those high-risk patients with 12 reinterventions, seven underwent OSR conversion, and four were treated with a second EVAR. Of those four, three required further intervention for type 1A endoleak, type III endoleak, and right limb thrombosis, respectively. Later on, two were repaired by OSR conversion and one by EVAR. The mean time until the second intervention was 777.25 days, and the third intervention's mean time was 140 days.

The most commonly used endografts were bifurcated Medtronic Talent (5) and AUI/bifurcated Medtronic Endurant (Santa Rosa, CA, USA) (28) and Unibody Endologix AFX and Powerlink (Endologix, Irvine, CA, USA) (12). Bifurcated Gore Excluder (W.L. Gore & Associates, Flagstaff, AZ, USA) (17), and Cordis Incraft (Cordis, a Johnson & Johnson company, Miami Lakes, FL, USA (14).

Type I A endoleak was primarily noticed in Medtronic Endurant grafts (50%), acute graft thrombosis in Cordis (33%), and type III endoleaks in Endologicx (16%). Gore graft had the lowest complication rate over the follow-up. We witnessed a higher incidence of chronic renal failure in endografts with suprarenal stents (10/63). Amongst the OSR group, 75% (*n* = 27/36) were repaired with silver Dacron tube graft, and (*n* = 9/36) were repaired by bifurcated graft with no difference in morbidity or mortality in short or long-term.

## Discussion

Surgical treatment is the mainstay of treatment for PR-AAA, and when patients are unsuitable for open surgical repair (OSR), endovascular techniques may be utilized (6). Endovascular repair of PR-AAA can include BEVAR, FEVAR, Ch-EVAR, or EVAR used outside of its instructions for use (IFU) and endografts, which are designed specifically for short neck (7–11). However, due to a suboptimal proximal sealing zone, PR-AAA repair confers an increased risk of graft migration, endoleak, and renal artery occlusion (9, 12).

Over two decades, from 2002 to 2023, advancements and insights in treating AAAs have significantly evolved. Beginning in 2002, our focus was on high-risk patients, demonstrating the superiority of EVAR over the best medical therapy (BMT) in reducing aneurysm-related mortality and offering cost-effective alternatives to OSR. By 2011, our attention shifted to pararenal AAAs, comparing outcomes between EVAR and OSR (27). Despite the differences in patient characteristics, EVAR emerged as a viable option with comparable long-term outcomes and greater cost-effectiveness. The landscape further transformed in 2019, reaffirming EVAR's superiority over OSR in perioperative morbidity and cost per quality-adjusted life year (QALY). However, by 2022, our understanding deepened, uncovering nuanced complications associated with specific graft designs and materials and highlighting the importance of ongoing research and refinement in endovascular techniques. This progression underscores the iterative nature of medical research, driving continuous improvement in patient outcomes and healthcare efficiency (27, 31–33).

In 2011, we analyzed 118 patients with pararenal AAAs, among whom 66 underwent OSR and 52 underwent pararenal EVAR (27). Patients undergoing pararenal EVAR had 15 cm of aortic neck and were older with higher comorbidity severity scores compared to OSR patients. OSR patients had larger aneurysm diameters.

There was no perioperative mortality in the pararenal EVAR group compared to 4.5% mortality among OSR patients. Pararenal EVAR also had lower 30-day morbidity compared to OSR. Three-year freedom from secondary intervention and all-cause survival were similar between the two groups. Pararenal EVAR was found to be cost-effective compared to OSR (27).

However, we witnessed that not many studies have addressed the unexplored aspect of kidney function and the occurrence of AKI in high-risk patients undergoing EVAR with short aortic necks, a population for whom EVAR is performed outside the recommended indications compared to OSR. Our study shed light on this challenging topic, providing valuable insights into the renal outcomes of these patients. Our findings contribute to a more comprehensive understanding of the risks and benefits associated with EVAR in unconventional scenarios, paving the way for tailored approaches to patient management and improved outcomes in this complex subset of AAA patients.

Studies have highlighted the significant incidence of AKI following suprarenal clamping during AAA repair (34–36). The incidence ranges from 26.5% to 37%, indicating the substantial risk associated with renal ischemia during the procedure. Despite efforts such as hypothermic renal protection techniques, AKI remains unchanged, underscoring the importance of refining surgical methods beyond mere interventions (34, 35).

Renal ischemia time correlates directly with intraoperative blood loss and AKI occurrence, emphasizing the need for precise perioperative management strategies. Identifying predictors of AKI can assist surgeons in optimizing patient care, potentially mitigating the risk of postoperative complications and mortality. This underscores the significance of meticulous surgical planning and execution to minimize ischemic insult to the kidneys during AAA repair (34–36).

An expeditious proximal anastomosis emerges as a pivotal factor in AAA repair, outweighing the emphasis on maintaining clamp position below a single renal artery. This suggests that suprarenal clamping might be the optimal approach to ensure efficient proximal anastomosis creation, emphasizing the importance of surgical technique over clamp positioning for improved outcomes (34–37).

Furthermore, choosing a proximal clamp site during AAA repair should prioritize anatomical considerations over perceived mortality benefits. Opting for the safest, distal-most level for proximal aortic clamping helps reduce cardiac morbidity and the risk of postoperative dialysis, reflecting a meticulous approach to minimize complications and enhance patient recovery. Efforts to keep the proximal clamp level as low as possible in patients with compromised renal function are crucial to mitigate renal morbidity and mortality risks associated with supra-celiac or suprarenal clamping (37–40).

We witnessed significantly higher AKI in the OSR group compared to the EVAR group. Zlatanovic et al. reported significantly higher AKI in the OSR group compared to the endovascular group (30.3% vs. 16.6%; *P* < 0.001) (41). A recent study at the five high-volume European academic centers showed similar outcomes with higher AKI frequency in the OSR group (40.7% vs. 24.8%; *p* = 0.006) (42). Dubois et al. found transient renal dysfunction of 37.3% after OSR of PR-AAA (43).

A meta-analysis of the incidence of EVAR of any kind causing renal impairment by Karthikesalingam et al. found that in the first year, 18% of patients experienced a clinically relevant change in renal function (11). At the same time, serum creatinine increased by 0.05 mg/dl at 30 days and 0.11 mg/dl in the first year, and creatinine clearance decreased by 5.65 ml/min at one month. Ultee et al. found a significant difference in renal dysfunction (baseline cr >1.5) in infrarenal AAA repair compared with AAA with hostile neck defined at JRAAAs and PRAAAs (2.3% vs. 9.5%, *P* < .001) (44).

Previous reports have shown a greater decline in renal function in EVAR (8.2 ml/min/1.73m^2^) at five years compared to OSR (7.4 ml/min/1.73m^2^) (15). This study also showed a more favourable 5-year difference for OSR (4.29 ml/min/1.73m^2^) and EVAR (6.63 ml/min/1.73m^2^).

Similarly, in terms of CKD, although there was no significant difference between the two groups, there was a significant change in mean creatinine from baseline to 5-year follow-up (0.024). The DREAM trial (45) showed a mean fall in eGFR 4.2 ml/min/1.73m^2^ post-EVAR at five years, while EVAR-1/EVAR-2 trial (14) showed a mean drop in eGFR of 1.13 ml/min/1.73m^2^ per year for EVAR and −1.00 ml/min/1.73m^2^ per year for the open repair groups, which is similar to the findings of this study (1.32 ml/min/1.73m^2^).

Regarding the LRVDL, there have been mixed reports concerning the effect of LRVDL on renal function. Some authors have reported a decline in renal function (46). Similarly, studies have shown that postoperative AKI requiring dialysis is associated with adverse outcomes, and the rate of dialysis could fall between 5% and 15% (47, 48). Our results showed that 27.8% of patients with LRVDL and 50% without LRVDL experienced an AKI (*P* = 0.382). Of particular note is the finding that patients who experienced AKI in the OSR group were not significantly affected by LRVDL. This finding highlights the complex interplay of factors contributing to renal dysfunction in the context of OSR for PR-AAA, warranting further investigation into the underlying mechanisms and potential strategies for mitigation.

Our perioperative mortality after OSR repair was 2.7% (1/36), in keeping with results reported by Malas *et al*. at 3.7% (49). Dubois et al. showed an overall mortality of 4.1%, although this increased to 9.5% in patients with post-op renal dysfunction (43). However, there was no perioperative mortality among the 63 patients who underwent EVAR. This finding is compared favorably with previous findings of 2% by Antoniou et al. (50) and Ultee et al. (44), who found a 30-day mortality of 6.6% in patients with complex neck anatomy.

Good surgical technique minimizes postoperative complications, particularly in procedures like open AAA repair. The location and efficiency of anastomosis play a crucial role in patient outcomes, regardless of whether it involves one or two renal arteries. Left renal vein transfixing emerges as a critical step, exposing the juxta renal area entirely and facilitating optimal anastomosis placement. Furthermore, postoperative care could be crucial. We routinely treat patients in the postoperative period with intravenous saline, N-acetylcysteine, mannitol 20%, intravenous prophylactic antibiotics, heparin, prostacyclin PGI2 analog, and the cell-saver (51).

This study sheds light on several key findings regarding the management and associated risks of AKI in OSR compared to EVAR. Although there was a higher incidence of AKI in the OSR group, surpassing rates reported in previous literature, endovascular renal events were comparable to other studies, suggesting consistency in outcomes across different cohorts.

Moreover, it underscores the utility of EVAR in managing PR-AAA, particularly in the postoperative period. Despite the higher incidence of AKI in the OSR group, the long-term follow-up revealed similar outcomes between the two groups, with EVAR demonstrating superiority in the immediate postoperative phase. This finding underscores the importance of considering short-term outcomes and long-term implications when choosing the optimal treatment approach for PR-AAA.

The recent UK COMPlex AneurySm Study (UK-COMPASS) (52) compares perioperative and midterm outcomes for OSR and EVAR for juxta-renal and complex neck aortic aneurysms. Complex AAAs are defined as aneurysms involving the reno-visceral segment without involving the thoracic aorta. Our results align with those of the UK COMPASS study, where open surgery was offered to a younger and healthier cohort of patients. The outcomes of OSR vs. EVAR for complex necks were comparable. There was no statistically significant difference in mortality or survival benefit between EVAR and OSR for juxta-renal, short-neck, or complex-neck aneurysms. However, the survival benefit of EVAR diminishes after 21 months and is 2.5 to 3 times less than that of OSR.

Juxta-renal OSR has the highest mortality in this anatomical group, while in high-risk patients, there is no difference in mortality between OSR and EVAR (52). For standard-risk patients, the survival benefit of EVAR is lost within months, and mortality is almost threefold higher than for OSR.

Our manuscript presents real-world data that is highly generalizable. We observed a high frequency of off-label EVAR, with one-third of procedures for short-neck and nearly two-thirds for complex-neck aneurysms.

Perioperative AKI and long-term outcomes was more common in OSR at 30 days, but long-term renal function did not differ significantly between OSR and EVAR. Most perioperative mortality occurred in the juxta-renal group, attributable to the level of aortic cross-clamping. For standard-risk patients, the open repair approach generally yields better outcomes beyond the first year, regardless of the aortic neck complexity. We confirmed that in high-risk patients, all surgical interventions result in somewhat similar outcomes and poor survival outlooks. This reinforces the need for careful patient selection and individualized treatment planning for complex AAAs.

A strength of this study is that there was no significant difference between age or comorbidities profile, and most of the OSR and EVAR were JR-AAAs. The standardization of AKI for patients undergoing AAA repair and the classification of PR-AAA and JR-AAA should be revisited, as there is no consensus in the literature regarding which definitions to use. A large-scale, long-term study is required to further elucidate the value of EVAR over OSR in this cohort of patients.

Given the current evidence and outcomes, it is crucial to prioritize vascular patients for timely treatment, utilizing the most appropriate surgical technique based on individual patient risk profiles and aneurysm characteristics. By doing so, we can improve patient outcomes, reduce perioperative risks, and ensure better long-term survival.

### Limitations

This study is limited as it is a single longitudinal study. Comparing the results of this study with those of others was difficult due to the use of several definitions for the JR-AAA, SR-AAA, and PR-AAA. This observation was also the case with AKI with *ad hoc* cut-off points and alternative criteria, including the Acute Kidney Injury Network classification (AKIN), kidney disease–improving global outcomes (KDIGO), and the Aneurysm Renal Injury Score (ARISe) were used (12, 16, 53).

### Need for randomized controlled trials (RCTs)

There is a need for RCTs to compare the two principal methods for complex AAA repair: OSR vs. complex BEVAR and FEVAR, as well as off-label EVAR with or without adjuncts. Patients with an AAA ≥5.5 cm and a neck <10 mm, or a complex neck unsuitable for on-label standard EVAR (e.g., conical neck, large diameter, excessive angulation, calcification, or thrombus), are ideal candidates for such studies.

## Conclusion

We found significantly higher AKI in the OSR group compared to the EVAR group, providing valuable insights into the management of PR-AAA and the associated risks of AKI. This observation highlights the need for individualized treatment strategies tailored to patient-specific factors and long-term outcomes. Further research is warranted to elucidate the optimal approach to PR-AAA repair and refine perioperative care protocols to minimize renal morbidity and optimize patient outcomes.

## Data Availability

The raw data supporting the conclusions of this article will be made available by the authors, without undue reservation.

## References

[1] Knott AW Kalra M Duncan AA Reed NR Bower TC Hoskin TL Open repair of juxtarenal aortic aneurysms (JAA) remains a safe option in the era of fenestrated endografts. J Vasc Surg. (2008) 47:695–701. 10.1016/j.jvs.2007.12.007 18272317

[2] Qvarfordt PG Stoney RJ Reilly LM Skioldebrand CG Goldstone J Ehrenfeld WK. Management of pararenal aneurysms of the abdominal aorta. J Vasc Surg. (1986) 3:84–93. 10.1067/mva.1986.avs0030084 3941485

[3] Crawford ES Beckett WC Greer MS. Juxtarenal infrarenal abdominal aortic aneurysm. Special diagnostic and therapeutic considerations. Ann Surg. (1986) 203:661–70. 10.1097/00000658-198606000-00011 3521511 PMC1251200

[4] Jeyabalan G Park T Rhee RY Makaroun MS Cho JS. Comparison of modern open infrarenal and pararenal abdominal aortic aneurysm repair on early outcomes and renal dysfunction at one year. J Vasc Surg. (2011) 54:654–9. 10.1016/j.jvs.2011.03.007 21620619

[5] Fairman RM Velazquez OC Carpenter JP Woo E Baum RA Golden MA Midterm pivotal trial results of the talent low profile system for repair of abdominal aortic aneurysm: analysis of complicated versus uncomplicated aortic necks. J Vasc Surg. (2004) 40:1074–82. 10.1016/j.jvs.2004.09.013 15622358

[6] Chiesa R Marone EM Brioschi C Frigerio S Tshomba Y Melissano G. Open repair of pararenal aortic aneurysms: operative management, early results, and risk factor analysis. Ann Vasc Surg. (2006) 20:739–46. 10.1007/s10016-006-9134-8 17072494

[7] Banno H Cochennec F Marzelle J Becquemin JP. Comparison of fenestrated endovascular aneurysm repair and chimney graft techniques for pararenal aortic aneurysm. J Vasc Surg. (2014) 60:31–9. 10.1016/j.jvs.2014.01.036 24560863

[8] Quatromoni JG Orlova K Foley PJ 3rd. Advanced endovascular approaches in the management of challenging proximal aortic neck anatomy: traditional endografts and the snorkel technique. Semin Intervent Radiol. (2015) 32:289–303. 10.1055/s-0035-1558825 26327748 PMC4540613

[9] Medical Advisory Secretariat. Fenestrated endovascular grafts for the repair of juxtarenal aortic aneurysms: an evidence-based analysis. Ont Health Technol Assess Ser. (2009) 9:1–51. PMC3377528 23074534

[10] Katsargyris A Oikonomou K Klonaris C Töpel I Verhoeven EL. Comparison of outcomes with open, fenestrated, and chimney graft repair of juxtarenal aneurysms: are we ready for a paradigm shift? J Endovasc Ther. (2013) 20:159–69. 10.1583/1545-1550-20.2.159 23581756

[11] Karthikesalingam A Bahia SS Patel SR Azhar B Jackson D Cresswell L A systematic review and meta-analysis indicates underreporting of renal dysfunction following endovascular aneurysm repair. Kidney Int. (2015) 87:442–51. 10.1038/ki.2014.272 25140912 PMC5590709

[12] Mehta RL Kellum JA Shah SV Molitoris BA Ronco C Warnock DG Acute kidney injury network: report of an initiative to improve outcomes in acute kidney injury. Crit Care. (2007) 11:R31. 10.1186/cc5713 17331245 PMC2206446

[13] Nana P Kouvelos G Brotis A Spanos K Giannoukas A Matsagkas M. The effect of endovascular aneurysm repair on renal function in patients treated for abdominal aortic aneurysm. C. Curr Pharm Des. (2019) 25:4675–85. 10.2174/1381612825666191129094923 31782360

[14] Brown LC Brown EA Greenhalgh RM Powell JT Thompson SG, UK EVAR Trial Participants. Renal function and abdominal aortic aneurysm (AAA): the impact of different management strategies on long-term renal function in the UK EndoVascular aneurysm repair (EVAR) trials. Ann Surg. (2010) 251:966–75. 10.1097/SLA.0b013e3181d9767c 20395842

[15] Saratzis A Bath MF Harrison S Sayers RD Mahmood A Sarafidis P Long-term renal function after endovascular aneurysm repair. Clin J Am Soc Nephrol. (2015) 10:1930–6. 10.2215/CJN.04870515 26487770 PMC4633790

[16] Khwaja A. KDIGO Clinical practice guidelines for acute kidney injury. Nephron Clin Pr. (2012) 120:179–84. 10.1159/000339789 22890468

[17] Jean-Claude JM Reilly LM Stoney RJ Messina LM. Pararenal aortic aneurysms: the future of open aortic aneurysm repair. J Vasc Surg. (1999) 29:902–12. 10.1016/s0741-5214(99)70218-1 10231642

[18] West CA Noel AA Bower TC Cherry KJ Jr Gloviczki P Sullivan TM Factors affecting outcomes of open surgical repair of pararenal aortic aneurysms: a 10-year experience. J Vasc Surg. (2006) 43:921–7. discussion 927-8. 10.1016/j.jvs.2006.01.018 16678684

[19] Boules TN Stanziale SF Chomic A Selzer F Tublin ME Makaroun MS. Predictors of diffuse renal microembolization following endovascular repair of abdominal aortic aneurysms. Vasc. (2007) 15:18–23. 10.2310/6670.2007.00006 17382050

[20] Chaikof EL Fillinger MF Matsumura JS Rutherford RB White GH Blankensteijn JD Identifying and grading factors that modify the outcome of endovascular aortic aneurysm repair. J Vasc Surg. (2002) 35:1061–6. 10.1067/mva.2002.123991 12021728

[21] Mehta M Valdés FE Nolte T Mishkel GJ Jordan WD Gray B One-year outcomes from an international study of the ovation abdominal stent graft system for endovascular aneurysm repair. J Vasc Surg. (2014) 59:65–73.e1-3. 10.1016/j.jvs.2013.06.065 23978572

[22] Aburahma AF Campbell JE Mousa AY Hass SM Stone PA Jain A Clinical outcomes for hostile versus favorable aortic neck anatomy in endovascular aortic aneurysm repair using modular devices. J Vasc Surg. (2011) 54:13–21. 10.1016/j.jvs.2010.12.010 21324631

[23] Walsh SR Tang T Gaunt ME Boyle JR. Contrast-induced nephropathy. J Endovasc Ther. (2007) 14:92–100. 10.1583/06-2035.1 17291156

[24] Wahlberg E Dimuzio PJ Stoney RJ. Aortic clamping during elective operations for infrarenal disease: the influence of clamping time on renal function. J Vasc Surg. (2002) 36:13–8. 10.1067/mva.2002.123679 12096250

[25] Samson RH Lepore MR Jr Showalter DP Nair DG Lanoue JB. Long-term safety of left renal vein division and ligation to expedite complex abdominal aortic surgery. J Vasc Surg. (2009) 50:500–4. 10.1016/j.jvs.2009.04.041 19595540

[26] Settembrini AM Aronici M Martelli E Casella F Martelli M Renghi A Is Mini-invasive surgery an alternative for the treatment of Juxtarenal aortic aneurysms? Ann Vasc Surg. (2022) 78:220–5. 10.1016/j.avsg.2021.06.014 34455043

[27] Sultan S Hynes N. Clinical efficacy and cost per quality-adjusted life years of pararenal endovascular aortic aneurysm repair compared with open surgical repair. J Endovasc Ther. (2011) 18:181–96. 10.1583/10-3072.1 21521058

[28] Bellomo R Ronco C Kellum JA Mehta RL Palevsky P, Acute Dialysis Quality Initiative workgroup. Acute renal failure—definition, outcome measures, animal models, fluid therapy and information technology needs: the second international consensus conference of the acute dialysis quality initiative (ADQI) group. Crit Care. (2004) 8:R204–12. 10.1186/cc2872 15312219 PMC522841

[29] Manjunath G Sarnak MJ Levey AS. Prediction equations to estimate glomerular filtration rate: an update. Curr Opin Nephrol Hypertens. (2001) 10:785–92. 10.1097/00041552-200111000-00009 11706306

[30] Kampmann JD Heaf JG Mogensen CB Mickley H Wolff DL Brandt F. Prevalence and incidence of chronic kidney disease stage 3–5 - results from KidDiCo. BMC Nephrol. (2023) 24:17. 10.1186/s12882-023-03056-x 36658506 PMC9849831

[31] Hynes N Sultan S. A prospective clinical, economic, and quality-of-life analysis comparing endovascular aneurysm repair (EVAR), open repair, and best medical treatment in high-risk patients with abdominal aortic aneurysms suitable for EVAR: the Irish patient trial. J Endovasc Ther. (2007) 14:763–76. 10.1583/07-2194.1 18052596

[32] Sultan S Acharya Y Soliman O Parodi JC Hynes N. TEVAR And EVAR, the unknown knowns of the cardiovascular hemodynamics; and the immediate and long-term consequences of fabric material on major adverse clinical outcome. Front Surg. (2022) 9:940304. 10.3389/fsurg.2022.940304 36111231 PMC9468223

[33] Sultan S Acharya Y Zayed O Elzomour H Parodi JC Soliman O Is the cardiovascular specialist ready for the fifth revolution? The role of artificial intelligence, machine learning, big data analysis, intelligent swarming, and knowledge-centered service on the future of global cardiovascular healthcare delivery. J Endovasc Ther. (2023) 30:877–84. 10.1177/15266028221102660 35695277 PMC10637093

[34] Yokoyama N Nonaka T Kimura N Sasabuchi Y Hori D Matsunaga W Acute kidney injury following elective open aortic repair with suprarenal clamping. Ann Vasc Dis. (2020) 13:45–51. 10.3400/avd.oa.19-00095 32273921 PMC7140154

[35] O'Donnell TFX Boitano LT Deery SE Clouse WD Siracuse JJ Schermerhorn ML Factors associated with postoperative renal dysfunction and the subsequent impact on survival after open juxtarenal abdominal aortic aneurysm repair. J Vasc Surg. (2019) 69:1421–8. 10.1016/j.jvs.2018.07.066 30477939

[36] Mehta A O'Donnell TFX Schutzer R Trestman E Garg K Mohebali J Evaluating proximal clamp site and intraoperative ischemia time among open repair of juxtarenal aneurysms. J Vasc Surg. (2022) 76:411–8. 10.1016/j.jvs.2022.01.126 35149161

[37] Grab J Krzyzaniak H Devrome A Moore R. Efficacy of cold renal perfusion protection for open complex aortic aneurysm repair: a meta-analysis. Canadian journal of surgery. Can J Surg. (2022) 65:E805–15. 10.1503/cjs.017821 36418066 PMC9710859

[38] Natour AK Kabbani L Rteil A Nypaver T Weaver M Lee A Cross-clamp location and perioperative outcomes after open infrarenal abdominal aortic aneurysm repair: a vascular quality initiative® review. Vascular. (2023) 31:199–210. 10.1177/17085381211067616 35435780

[39] Zhang K Zheng H Hu Z Liang Z Hao Y Chen Z. Endovascular repair versus open surgical repair for Complex abdominal aortic aneurysms: a systematic review and meta-analysis. Ann Vasc Surg. (2023) 93:355–68. 10.1016/j.avsg.2022.06.102 35926793

[40] Rosenfeld ES Macsata RA Nguyen BN Lala S Ricotta JJ Pomy BJ Thirty-day outcomes of open abdominal aortic aneurysm repair by proximal clamp level in patients with normal and impaired renal function. J Vasc Surg. (2021) 73:1234–1244.e1. 10.1016/j.jvs.2020.08.122 32890718

[41] Zlatanovic P. Davidovic L. Mascia D. Ancetti S. Yeung K.K. Jongkind V. (2024). Acute kidney injury in patients undergoing endovascular or open repair of juxtarenal or pararenal aortic aneurysms. J Vasc Surg*.* 79:1347–1359.e3. 10.1016/j.jvs.2024.02.021 38395093

[42] Zlatanovic P Mascia D Ancetti S Yeung KK Graumans MJ Jongkind V Short term and long term clinical outcomes of endovascular versus open repair for juxtarenal and pararenal abdominal aortic aneurysms using propensity score matching: results from juxta- and pararenal aortic aneurysm multicentre European study (JAMES). Eur J Vasc Endovasc Surg. (2023) 65:828–36. 10.1016/j.ejvs.2023.02.070 36858252

[43] Dubois L Durant C Harrington DM Forbes TL Derose G Harris JR. Technical factors are strongest predictors of postoperative renal dysfunction after open transperitoneal juxtarenal abdominal aortic aneurysm repair. J Vasc Surg. (2013) 57:648–54. 10.1016/j.jvs.2012.09.043 23312936

[44] Ultee KHJ Zettervall SL Soden PA Darling J Verhagen HJM Schermerhorn ML. Perioperative outcome of endovascular repair for complex abdominal aortic aneurysms. J Vasc Surg. (2017) 65:1567–75. 10.1016/j.jvs.2016.10.123 28216344 PMC5438879

[45] de Bruin JL Vervloet MG Buimer MG Baas AF Prinssen M Blankensteijn JD Renal function 5 years after open and endovascular aortic aneurysm repair from a randomized trial. Br J Surg. (2013) 100:1465–70. 10.1002/bjs.9280 24037566

[46] Huber D Harris JP Walker PJ May J Tyrer P. Does division of the left renal vein during aortic surgery adversely affect renal function? Ann Vasc Surg. (1991) 5:74–9. 10.1007/BF02021783 1997081

[47] Kashyap VS Cambria RP Davison JK L'Italien GJ. Renal failure after thoracoabdominal aortic surgery. J Vasc Surg. (1997) 26:949–57. 10.1016/s0741-5214(97)70006-5 9423709

[48] Schepens MA Defauw JJ Hamerlijnck RP Vermeulen FE. Risk assessment of acute renal failure after thoracoabdominal aortic aneurysm surgery. Ann Surg. (1994) 219:400–7. 10.1097/00000658-199404000-00011 8161266 PMC1243157

[49] Malas M Arhuidese I Qazi U Black J Perler B Freischlag JA. Perioperative mortality following repair of abdominal aortic aneurysms: application of a randomized clinical trial to real-world practice using a validated nationwide data set. JAMA Surg. (2014) 149:1260–5. 10.1001/jamasurg.2014.275 25337871

[50] Antoniou GA Georgiadis GS Antoniou SA Kuhan G Murray D. A meta-analysis of outcomes of endovascular abdominal aortic aneurysm repair in patients with hostile and friendly neck anatomy. J Vasc Surg. (2013) 57:527–38. 10.1016/j.jvs.2012.09.050 23265584

[51] Beirne C Hynes N Sultan S. Six years’ experience with prostaglandin I2 infusion in elective open repair of abdominal aortic aneurysm: a parallel group observational study in a tertiary referral vascular center. Ann Vasc Surg. (2008) 22:750–5. 10.1016/j.avsg.2008.08.036 18992665

[52] Vallabhaneni SR Patel SR Campbell B Boyle JR Cook A Crosher A Editor’s choice—comparison of open surgery and endovascular techniques for juxtarenal and complex neck aortic aneurysms: the UK COMPlex AneurySm study (UK-COMPASS)—peri-operative and midterm outcomes. Eur J Vasc Endovasc Surg. (2024) 67:540–53. 10.1016/j.ejvs.2024.02.037 38428672

[53] Twine CP Boyle JR. Renal dysfunction after EVAR: time for a standard definition. J Endovasc Ther. (2013) 20:331–3. 10.1583/12-4104C.1 23731305

